# Timing of adiposity rebound in children with congenital hypothyroidism diagnosed by newborn screening and treated with Levothyroxine

**DOI:** 10.1186/s13052-025-02099-0

**Published:** 2025-08-08

**Authors:** Cecilia Lugarà, Domenico Corica, Giorgia Pepe, Maria Francesca Messina, Mariella Valenzise, Giuseppina Zirilli, Letteria Anna Morabito, Alessandra Li Pomi, Malgorzata Gabriela Wasniewska, Tommaso Aversa

**Affiliations:** 1 Department of Human Pathology of Adulthood and Childhood, University of Messina, Via Consolare Valeria, Messina, 98124 Italy; 2 Pediatric Unit, “G. Martino” University Hospital, Via Consolare Valeria, Messina, 98124 Italy

**Keywords:** Congenital hypothyroidism, Neonatal screening, Levothyroxine, Adiposity rebound, Early adiposity rebound, Childhood obesity

## Abstract

**Background:**

Children with congenital hypothyroidism (CH) are at increased risk of developing childhood obesity. Moreover, an adiposity rebound (AR) that occur early is strongly linked with future obesity. The aims of our study were to explore the timing of AR, to identify factors affecting AR and correlation between age and body mass index (BMI) at AR and BMI at eight years of age in children with CH.

**Methods:**

A retrospective, observational study was conducted on Caucasian children with CH. For each child, BMI growth curve was constructed, AR was identified and compared with the Italian national and regional references.

**Results:**

Sixty-nine patients (44 females) were recruited. The age at AR was 3.4 ± 1.2 vs. 4.5 years of the comparison population (*p* < 0.001) in females and 3.4 ± 1.4 vs. 5.0 years of the comparison population (*p* < 0.001) in males with CH. Age at AR showed a significant negative correlation with BMI at 8 years (*r* = − 0.274, *p* < 0.039). BMI at AR and BMI at 8 years of age correlated positively (*r* = 0.460, *p* < 0.001). The prevalence of obesity/overweight at 8 years of age was 30%. BMI at 8 years of age was 22.4 ± 1.8 kg/m^2^ in overweight/obese subgroup and 16.9 ± 1.8 kg/m^2^ in normal BMI subgroup (*P* 0.001).

**Conclusion:**

Children with CH showed earlier AR compared to Italian national references. The higher risk of obesity in patients with early AR is supported by the association between age, BMI at AR, and BMI at eight years.

## Background

Congenital hypothyroidism (CH) is a disease characterized by a defect in production and/or secretion of thyroid hormone (TH). TH is essential to guarantee the child’s healthy neurodevelopment and growth. CH can be caused by a defect in the thyroid gland (primary CH) or in the pituitary/hypothalamus (central CH). Primary CH may result from either impaired thyroid development or failure of TH production. The defect in thyroid development is called thyroid dysgenesis which includes a spectrum of abnormalities such as agenesis, ectopy, or hypoplasia of the thyroid gland. The defect in TH production is called dyshormonogenesis, caused by genetic deficiency in enzymes involved in the hormonal biosynthesis [1–3]. CH is the most common cause of endocrinopathy in the newborn, with a current incidence of between 1:1000 and 1:3000. This variable incidence depends on the screening TSH cut off value, but also environmental, ethnic, and genetic factors should be considered [4–7]. Neonatal screening programs were established in the 1970 and are nowadays available in 30% of countries worldwide. In Italy, CH is detected through a neonatal screening program since 1977. TSH levels are the basis of the screening program; nevertheless, the cut-offs may be different in each region. When a patient is diagnosed with CH, a replacement treatment with levothyroxine must be started in order to prevent intellectual disability [1–7]. Children with CH, treated with levothyroxine within the first month of life at the appropriate dosage, show height growth and pubertal development comparable to the general population [8–11]. Regarding body mass index (BMI), an increase in overweight and obesity during childhood and young adulthood has been reported in these patients, despite adequate levothyroxine treatment. However, there are no statistically significant changes in BMI between CH patients who present with thyroid dysgenesis and those who present with dyshormonogenesis [10, 12–17]. The underlying reason for the increased risk of overweight and obesity in this population remain unclear. However, several studies have revealed that, among the different parameters examined, children with CH experienced an earlier onset of adiposity rebound (AR) than the general population [12–14].

AR refers to the second increase in a child’s BMI, typically occurring between the ages of 6 and 8 years. During the first year of life, the BMI naturally rises, then progressively decreases to a minimum value, increasing again during this rebound period [18]. Many different methods are used to determine the timing of AR. Comparing different strategies - visual estimation of individual BMI curves and identification using polynomial models - visual estimation has emerged as the most effective technique for accurately capturing the physiological underpinnings of AR. This is a straightforward method that permits rapid identification of the nadir point of AR [19]. Early AR refers to AR that happens before 6 years of age. An elevated risk of overweight and obesity in childhood and early adulthood is linked to early AR. Early AR also raises the risk of cardiometabolic consequences, including hypertension, dyslipidemia, elevated atherogenic index, and type 2 diabetes [18, 20–22]. According to the information previously mentioned, children with CH who experience early AR are consequently more likely to be overweight or obese during childhood and early adulthood, with potential cardio-metabolic complications.

The first aim of this study was to evaluate the timing of the AR and the potential factors affecting it in children with CH diagnosed by neonatal screening and treated with regular, appropriate replacement therapy. A second aim, given the established association between early AR and an increased risk of overweight or obesity during childhood and adulthood, was to explore the relationship between the age and BMI at AR and the BMI at eight years of age in this population.

## Methods

A retrospective, single-center, observational study was conducted, to investigate the timing of AR, in children with CH followed at the Pediatric Endocrinology Centre of the University Hospital “G. Martino” of Messina, Italy. All the participants enrolled in this study were children of Caucasian ethnicity, predominantly from South Italy. The time of the follow-up considered was from the time of the diagnosis until the age of eight years.

The inclusion criteria were (a) being born appropriate for gestational age; (b) have been diagnosed with CH through newborn screening; (c) have begun levothyroxine therapy during the first month of life.

The exclusion criteria were (a) have been admitted to a neonatal critical care unit during the first month of life; (b) have been diagnosed with transient CH; (c) suffering a chronic disease that restricted growth during the follow up; (d) failing to attend routine follow-up checkups.

The diagnosis of CH was done through a newborn screening procedure. Each child who screened positive for CH was referred to the Pediatric Endocrinology Centre for the first visit. Blood sample was collected to confirm CH by measuring serum TSH and FT4 levels. All patients started replacement therapy with levothyroxine, during the first month of life, at an initial mean dose of 9.2 ± 3.1 µg/kg/day (median value 9.9 µg/kg/day; interquartile range (IQR) 7.6–11.5 µg/kg/day), depending on the severity of the initial hypothyroidism, according to the European and American guidelines [1, 2]. During the follow-up, levothyroxine dosage was adjusted according to thyroid function.

In all patients with thyroid in situ, the diagnosis of CH was reevaluated at a median age of 3 years through a 30 days trial off thyroxine, followed by serum TSH and FT4 assay.

The initial cohort included 95 patients. 26 patients were excluded due to transient hypothyroidism or hyperthyreotropinemia (14), chronic diseases that affect normal growth (i.e. acute leukemia, celiac disease, GH deficiency) (5), or because they were lost during the follow-up (7). Sixty-nine patients with CH were included in this study (44 females and 25 males). The population was subdivided into two subgroups based on: (a) the CH etiology (dysgenesis vs. dyshormonogenesis) (b) the BMI z-score value at 8 years of age, according to Italian national and regional (Suth -Italy) references (overweight/obesity vs. normal weight) [23].

Patients who had a thyroid gland in normal anatomical location, of normal size, and without ecostructural, or other abnormalities were all classified as having thyroid dyshormonogenesis. Patients who presented with agenesis, hypoplasia, ectopia, or morphological and structural thyroid anomalies were classified as having thyroid dysgenesis.

Patients with a BMI z- score at or above + 1 standard deviation were classified as overweight. Patients with a BMI z- score at or above + 2 standard deviations were classified as obese. Patients with a BMI z- score between + 1 and– 2 standard deviations were classified as normal weight. In this population, no patients were underweighting at 8 years of age according to BMI z- score.

After the first diagnostic visit, patients attended the outpatient clinic every 2–3 months all through the first year of life, every 4 months during the second and third year of life, and then at intervals of 6 months until 8 years of age. Auxological parameters, laboratory results, instrumental investigations, and treatment data were recorded at each follow-up examination. Height measurements until age 2 were done in a supine position using Harpenden infantometer, and after that in a standing position using Harpenden stadiometer. Weight was recorded using a mechanical column scale with sliding counterweights. The BMI value was calculated using the formula weight (kg)/height (m^2^). A BMI growth curve was created for each child, and the AR point was identified using a graphic method. When BMI rises after the curve’s low point at a rate of at least 0.1 kg/m^2^, this is referred to as AR [19]. The auxological data concerning our population, subdivided by sex, were compared with the standards provided by the Italian national and regional (Suth Italy) growth charts for height, weight and BMI (2 to 20 years old) [23].

### Statistical method

Numerical variables were expressed as mean, SD, median and interquartile range (Q1-Q3); categorical variables were expressed as absolute frequencies and percentage. Non-parametric approach was used since the numerical variables were not normally distributed, such as verified by the Kolmogorov-Smirnov test. The one sample Wilcoxon signed rank test was used to compare BMI of children with CH at the different ages with the Italian national and regional references for BMI. The Mann Whitney test was applied with reference to numerical parameters to identify possible significant differences between the different groups of subjects divided according to the etiology (dysgenesis vs. dyshormonogenesis) and to BMI at 8 years of age (normal weight vs. obesity/overweight). The Spearman correlation test was applied to assess the relationship between age and BMI at AR and BMI at 8 years, and between the age at AR and thyroid function as well as levothyroxine posology at the time of CH diagnosis.

Statistical analyses were performed using IBM SPSS for Windows, Version 22 (Armonk, NY, IBM Corp.). A *p*-value lower than 0.05 was considered statistically significant.

## Results

### Characteristics of the population

Sixty-nine patients with CH were enrolled: 44 females and 25 males. Based on the aetiology of CH, the population was categorised into two subgroups: patients with thyroid dysgenesis and patients with thyroid dyshormonogenesis. Furthermore, we distinguished between subgroups of overweight/obese and normal-weight children at 8 years of age, based on BMI z-score values according to national growth charts. Table 1 shows the clinical and demographic characteristics of all CH patients as well as those of the different subgroups. Mean gestational age was 38.2 ± 2.4 weeks, while mean birth weight and mean birth length were respectively 3.0 ± 0.6 kg, and 47.8 ± 3.3 cm. Levothyroxine treatment was started at a mean age of 24 ± 9 days of life, with a mean initial dose of 9.2 ± 3.1 µg/kg/day (median value 9,9 µg/kg/day; IQR 7,6–11,5 µg/kg/day). The mean value of TSH at diagnosis was 321 ± 901.7 mUI/mL, and the mean value of FT4 was 15.5 ± 10.8 pmol/L.

**Table 1:** Demography and clinical characteristics of CH patients

	All	Dysgenesis	Dyshormonogenesis	*p*- value	Normal weight at 8 years		Overweight / Obesity at 8 years		*p*- value
N. of patients (percentage)	69 (100)	47 (68)	22 (32)	−	42 (70)		18 (30)		−
Gestational age (weeks)	38.2 ± 2.4 39.0; 37.0–40.0	38.6 ± 2.5 39.5; 37.0–40.0	37.6 ± 2.2 38.0; 36.5–39.0	0.041	38.7 ± 2.4 39.0; 38.0–40.0		38.1 ± 2.4 38.0; 38.0–40.0		0.206
Birth Weight (kg)	3.0 ± 0.6 3.1; 2.7–3.3	3.0 ± 0.6 3.2; 2.8–3.3	2.9 ± 0.6 2.8; 2.6–3.4	0.499	3.0 ± 0.6 3.2; 2.7–3.3		3.0 ± 0.5 3.0; 2.8–3.3		0.779
Birth Length (cm)	47.8 ± 3.3 48.0; 47.0–50.0	47.7 ± 3.4 48.0; 46.0–50.0	48.1 ± 3.3 48.5; 47.0–51.0	0.732	48.1 ± 3.4 49.0; 47.5–50.0		48.1 ± 2.8 48.0; 47.0–50.0		0.575
Age at CH diagnosis (days)	24 ± 9	23 ± 7	26 ± 11	0.873	24 ± 9		26 ± 8		0.455
TSH at CH diagnosis (mcrUl/mL)	321 ± 902 50; 17–292	455 ± 1099 100; 35–483	78 ± 176 25.4; 9.2–64	0.009	395.7 ± 1135 52.0; 11.3–430.0		255.6 ± 385.9 39.9; 35.7–381.0		0.634
FT4 at CH diagnosis (pmol/L)	15.5 ± 10.8 13.5; 7.5–17.7	16.6 ± 12.6 14.3; 7.5–21.3	13.8 ± 7.4 12.2; 9.2–17.0	0.762	19.4 ± 12.2 15.4; 11.8–24.2		13.6 ± 8.4 13.9; 7.7–15.9		0.258
L-T4 dose at diagnosis (µg /kg/day)	9.2 ± 3.1 9.9; 7.6–11.5	9.4 ± 2.7 9.9; 7.8–10.8	8.7 ± 4.0 9.7; 3.7–12.3	0.860	8.5 ± 3.1 8.9; 7.2–10.1		10.4 ± 2.7 10.6; 10.2–12.4		0.012
Age at AR (years)	3.4 ± 1.2 3.3; 2.5–4.2	3.3 ± 1.3 3.1; 2.2–4.1	3.5 ± 1.2 3.7; 2.5–4.4	0.401	3.4 ± 1.3 3.3; 2.4–4.4		3.1 ± 1.3 3.0; 2.2–4.1		0.356
BMI at AR (kg/m^2^)	15.9 ± 1.5 15.9; 15.2–16.9	16.1 ± 1.6 15.9; 15.3–17.4	15.6 ± 1.2 15.7; 14.8–16.4	0.208	16.3 ± 5.0 15.6; 15.0–16.2		16.7 ± 1.3 16.5; 15.4–18.0		0.028
TSH at AR (mcrUl/mL)	2.9 ± 4.1 2.1; 0.6–3.8	3.2 ± 4.9 2.1; 0.3–4.4	2.3 ± 1.6 2.2; 1.4–3.2	0.978	2.2 ± 2.2 1.8; 0.3–3.4		3.0 ± 2.5 2.5; 1.4–4.2		0.131
FT4 at AR (pmol/L)	17.6 ± 11.3 18.3; 16.5–21.9	17.9 ± 13.3 18.3; 15.5–21.7	17.1 ± 5.8 16.8; 14.8–20.2	0.383	20.9 ± 12.3 18.1; 16.2–22.4		19.9 ± 3.1 19.5; 17.8–23.0		0.374
L-T4 dose at AR (µg /kg/day)	3.4 ± 1.2 3.3; 2.6–4.1	3.7 ± 1.0 3.6; 3.1–4.3	2.6 ± 1.3 2.4; 1.7–3.0	< 0.001	3.6 ± 1.2 3.5; 2.9–4.5		3.4 ± 1.2 3.3; 2.5–3.9		0.333
BMI at 8 years (kg/m^2^)	18.6 ± 3.1 18.3; 16.3–20.9	18.7 ± 2.8 18.5; 16.4–20.5	18.4 ± 3.5 17.9; 15.9–21.6	0.579	16.9 ± 1.8 17.1; 15.8–18.5		22.4 ± 1.8 22.1; 21.2–22.8		< 0.001
TSH at 8 years (mcrUl/mL)	2.4 ± 1.7 2.0; 1.0–4.0	2.4 ± 1.8 2.0; 0.8–4.3	2.6 ± 1.2 2.2; 1.7–3.5	0.419	2.6 ± 1.7 2.3; 1.0–4.3		2.4 ± 2.1 2.0; 0.9–3.6		0.601
FT4 at 8 years (pmol/L)	18.7 ± 4.3 17.8; 16.0–21.4	19.5 ± 4.5 18.9; 16.3–22.2	16.4 ± 2.9 16.1; 15.1–17.0	0.011	18.8 ± 4.0 17.9; 15.8–22.1		18.3 ± 5.2 17.1; 16.1–19.4		0.638
L-T4 dose at 8 years (µg /kg/day)	2.3 ± 0.7 2.3; 1.9–2.7	2.5 ± 0.6 2.6; 2.0–3.0	1.7 ± 0.5 1.9; 1.2–2.2	< 0.001	2.5 ± 0.6 2.5; 2.0–3.0		1.9 ± 0.6 1.9; 1.4–2.4		0.006

Demographic and clinical characteristics (mean ± SD, median value and interquartile range (Q1-Q3)) of the entire population and of the subgroups according to congenital hypothyroidism etiology and according to BMI z-score at 8 years

CH: congenital hypothyroidism; L-T4: levothyroxine; AR: adiposity rebound; BMI: body mass index

When the BMI z-score of children with CH was evaluated up to the age of eight, it showed that, both males and females, had mean BMI z-score above + 0.0 SDS from the two years of life [Table 2].

**Table 2:** Mean and SD BMI of children with CH compared with Italian National and regional (South Italy) references

Girls with CH		References (Girls)		*p*- value		Boys with CH		References (Boys)		*p*- value	
**Age** **(Years)**	**BMI** **(kg/m2)**	**BMI 50° p whole Italy** **(kg/m2)**	**BMI 50° p South Italy** **(kg/m2)**	***CH vs. Whole Italy references***	***CH vs. South Italy references***	**Age** **(Years)**	**BMI** **(kg/m2)**	**BMI 50° p whole Italy** **(kg/m2)**	**BMI 50° p South Italy** **(kg/m2)**	***CH vs. Whole Italy references***	***CH vs. South Italy references***
0.13 ± 0.15	14.18 ± 1.38	------	------			0.19 ± 0.19	15.02 ± 1.78	------	------		
0.53 ± 0.13	17.49 ± 1.63	------	------			0.54 ± 0.09	17.59 ± 2.27	------	------		
1.03 ± 0.13	17.47 ± 1.39	------	------			1.05 ± 0.14	17.35 ± 1.57	------	------		
1.58 ± 0.18	17.12 ± 1.39	------	------			1.52 ± 0.1	16.95 ± 1.67	------	------		
2.07 ± 0.17	16.6 ± 1.29	16.1	16.1	0.0416	0.0416	2.06 ± 0.13	16.87 ± 1.74	16.5	15.8	*ns*	0.0057
2.57 ± 0.17	16.64 ± 1.18	15.8	15.9	0.0004	0.0012	2.55 ± 0.14	16.78 ± 1.81	16.1	15.8	*ns*	0.0152
3.1 ± 0.13	16.57 ± 1.16	15.7	15.8	0.0001	0.0002	3.1 ± 0.14	17.32 ± 2.01	15.9	15.7	0.0051	0.0019
3.6 ± 0.14	16.67 ± 1.04	15.6	15.8	< 0.001	0.0000	3.63 ± 0.16	17.06 ± 2.57	15.8	15.7	*ns*	0.0313
4.14 ± 0.15	16.58 ± 1.39	15.6	15.8	0.0001	0.0012	4.15 ± 0.12	16.83 ± 2.05	15.8	15.8	0.0395	0.0395
4.58 ± 0.16	16.58 ± 1.44	15.7	15.8	0.0009	0.0027	4.63 ± 0.12	16.46 ± 2.08	15.8	15.8	*ns*	*ns*
5.09 ± 0.15	16.73 ± 1.4	15.8	15.9	0.0002	0.0009	5.1 ± 0.18	17.74 ± 2.9	15.8	15.9	0.0087	0.0096
5.63 ± 0.15	16.81 ± 1.64	15.9	16.0	0.0010	0.0039	5.66 ± 0.13	17.74 ± 1.86	15.9	16.0	0.0242	0.0298
6.13 ± 0.16	17.02 ± 2.0	16.1	16.1	0.0120	0.0120	6.17 ± 0.13	18.38 ± 3.4	16.0	16.1	0.0112	0.0176
6.62 ± 0.15	16.94 ± 1.91	16.2	16.2	0.0320	0.0320	6.64 ± 0.14	18.602 ± 3.72	16.2	16.3	0.0279	0.0311
7.13 ± 0.18	17.37 ± 2.24	16.4	16.4	0.0135	0.0135	7.09 ± 0.18	18.9 ± 3.48	16.3	16.5	0.0139	0.0198
7.63 ± 0.16	17.91 ± 2.43	16.6	16.6	0.0030	0.0030	7.62 ± 0.18	19.3 ± 3.43	16.5	16.7	0.0146	0.0199
8.20 ± 0.17	18.13 ± 2.63	16.9	16.9	0.0073	0.0073	8.17 ± 0.17	19.84 ± 3.57	16.8	16.9	0.0097	0.0113

CH: congenital hypothyroidism; BMI: body mass index

Girls with CH consistently exhibited mean BMI values above the 50th percentile of Italian standards both for whole Italy and also for South Italy, at all follow-up visits, beginning at age 2 years until the age of 8 years (all comparisons, *p* < 0.05) [Table 2]; similarly, boys with CH showed mean BMI values significantly higher compared to the 50th percentile of Italian standards, both for whole Italy and for South Italy, starting at age 5 years and at every follow up visit until the age of 8 years (all comparisons, *p* < 0.05) [Table 2].

### Adiposity rebound

**Fig. 1:**
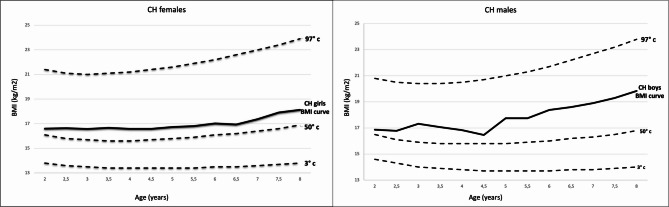
BMI growth curves of CH patients, grouped by sex, compared with Italian national references. (**a**) BMI growth curve for girls with CH compared with national references; (**b**) BMI growth curve for boys with CH compared with national references

The BMI trend in children with CH and in the national comparison population was graphically represented by the matching BMI growth curves for girls and boys [Fig. 1a and 1]. These curves were created using the data presented in Table 2 and the Italian national references.

Children with CH, regardless of gender, exhibited an average BMI above the 50th percentile for the reference population from 2 until 8 years of age.

In this cohort of children with CH, the mean age at first BMI peak was 0.9 ± 0.4 years and the mean BMI value was 18.0 ± 1.6 kg/m^2^ at first peak.

The mean age at the AR was 3.4 ± 1.2 years while the mean BMI value at AR was 15.9 ± 1.5 kg/m^2^. The mean dose of levothyroxine at the AR was 3.4 ± 1.2 µg/kg/day (median value 3.3 µg/kg/day; IQR 2.6–4.1 µg/kg/day).

The CH population was stratified by gender and compared to BMI national references standards.

The analysis showed that AR occurred earlier in both CH boys and CH girls than national references.

The females with CH experienced AR at mean age of 3.4 ± 1.2 years, significantly earlier than girls in the national reference population, who had a mean AR age of 4.5 years (*p* < 0.001) and the girls in the regional reference population, who had mean AR age of 5 years (*p* < 0.001). The mean BMI value at AR was 16.6 ± 4.7 kg/m^2^. The mean levothyroxine dose administered at the time of AR was 3.3 ± 1.1 µg/kg/day (median 3.3 µg/kg/day; IQR 2.7–4.1 µg/kg/day).

The males with CH had an average AR age of 3.4 ± 1.4 years, whereas significantly earlier than boys in the national reference population, who had a mean AR age of 5.5 years (*p* < 0.001) and in the regional reference population, who had mean AR age of 4.5 years (p 0.002). The mean BMI value at AR was 16.0 ± 1.8 kg/m^2^. The mean levothyroxine dose administered at AR was 3.4 ± 1.4 µg/kg/day (median 3.3 µg/kg/day; IQR 2.5–4.3 µg/kg/day).

No significant differences were observed between males and females with CH regarding age, BMI median value and posology of levothyroxine at the time of AR.

**Fig. 2:**
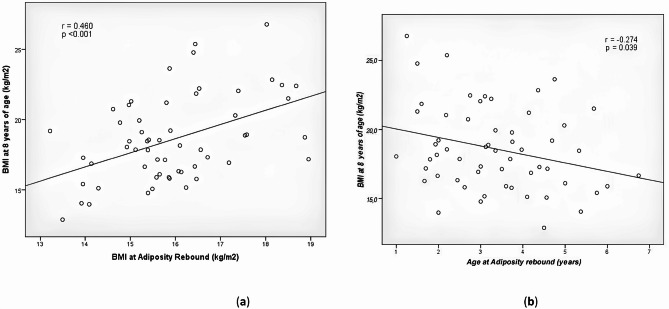
Correlation analysis between BMI at 8 years of age, age and BMI at the time of AR. (**a**) correlational analysis between BMI at AR and BMI at eight years of age; (**b**) correlational analysis between age at AR and BMI at eight years of age

### Factors potentially associated with early AR

In children with CH, the time of the AR did not significantly correlate with either the initial thyroid function (rho = 0.517, *p* = 0.126) or the levothyroxine beginning dose (rho = -0.080, *p* = 0.564). Regarding the etiology of CH, thyroid dysgenesia was seen in 47 children (68%), whereas thyroid dyshormonogenesis was observed in 22 children (32%). In both groups, AR was earlier than in general population (in children with thyroid dyshormonogenesis was at 3.5 ± 1.2 years, whist in children with thyroid dysgenesia at 3.3 ± 1.3 years). At the time of AR, the dysgenesis group received a higher mean levotiroxine dosage compared to the dyshormonogenesis group (3.7 ± 1.0 vs. 2.6 ± 1.3 µg/kg/day (*p* < 0.001)). Between two groups, there were no differences in age at AR, BMI at AR, TSH and FT4 value at AR and BMI mean value at age 8 years.

### Early AR in children with CH and risk of future obesity

The BMI z-score at age 8 years was used to divide the population into two subgroups: normal-weight and overweight/obese.

The prevalence of overweight/obesity at 8 years of age in children with CH was 30%. The mean BMI at 8 years of age was 22.4 ± 1.8 kg/m^2^ in the overweight/obese subgroup and 16.9 ± 1.8 kg/m^2^ in the normal weight subgroup (*p* < 0.001). Among the CH patients classified as overweight/obese, the distribution was equal between males and females (50% each gender). The mean levothyroxine dose administered at 8 years of age was 2.3 ± 0.7 µg/kg/day (median 2.3 µg/kg/day; IQR 1.9–2.7 µg/kg/day), whit a statistically difference between the normal-weight and overweight/obese groups (*p* = 0.006). Comparison of the two groups revealed that the mean levothyroxine dosage at the start of treatment was higher in the overweight/obese group compared to the normal-weight group ( 10.4 ± 2.7 vs. 8.5 ± 3.1 µg/kg/day, *p* = 0.012). When comparing the mean BMI values at AR for the two groups, CH children who were overweight/obese at the age of 8 had a higher mean BMI value at AR than the CH children who were normal weight at that age (BMI 16.7 ± 1.3 kg/m^2^ vs. 16.3 ± 5.0 kg/m^2^; *p* = 0.028). Analysis of the other features showed no significant differences between the two groups [Table 1]. No significant differences were observed between males and females with CH at 8 years of age in term of BMI median value and posology of levothyroxine.

Finally, BMI at the age of 8 years showed a positive correlation with the mean value of BMI at the AR (*p* < 0.001, *r* = 0.460) and a negative correlation with the age at AR (*p* value 0.039, *r* = -0.274) [Fig. 2a and b].

## Discussion

CH is the most common endocrinopathy in newborns and it was a cause of cognitive impairment and poor growth before the introduction of the neonatal screening program [1, 2, 24]. Despite the negative impact of TH deficiency on linear growth, growth velocity, and skeletal maturation, initiation of levothyroxine therapy within the first month of life and at an appropriate dosage, promote regular growth trajectories, pubertal progression, and the achievement of normal adult stature [10, 11, 24–27].

Whit regard to BMI growth trend in children with CH, this analysis revealed that, from two years of life, both male and female patients consistently exhibited BMI values above the 50th centile of the national reference standards. The literature corroborates these observations. Salerno et al. reported that the BMI was above the 95th percentile up to the age of 6 years, fluctuating between the 50th and 85th percentiles in the years that followed [9]. Grant D.B. showed that the average BMI of children with severe or mild congenital hypothyroidism was significantly higher than that of healthy French children at all ages [8]. Chen et al. described that, in comparison to the reference population, the BMI was above the 50th percentile from 2 to 10 years in girls with CH and from 2 to 4 years in boys with CH [14]. In a sample of 18 Bavarian children with CH at the age of 5.5 years, Arenz et al. found a median BMI between 13.7 and 21.5 kg/m^2^, corresponding to the 75th percentile of the Bavarian references [15]. Sun Q. et al., in a population study of Chinese children, showed that children with CH had a significantly higher risk of being overweight or obese between the ages of 0.5 and 6 years (*p* < 0.05) than controls who were healthy Chinese children [16]. Finally, in a large observational study of 1012 French CH patients, Leger et al. presented a higher percentage of overweight or obese subjects compared to the general French population (22.8 vs. 15.7%) (*p* < 0.001) [17].

To date, no clear explanation has been provided for the observation that children with CH, who are euthyroid, have a higher BMI than the reference population at nearly all ages. It is hipothyzed that the thyroid gland could play a fundamental role in the regulation of the energy metabolism and maintaining a healthy body weight. In fact, it is established that TH influence energy expenditure by regulating cellular metabolism, thermogenesis and basal metabolic rate, while TSH is involved in thermoregulatory mechanisms, regulates satiety and appetite by suppressing appetite, and modulates lipid accumulation by controlling lipolysis and lipogenesis. In a retrospective study on four hundred ninety-one Caucasian euthyroid obese children it was highlighted how TSH was positively correlated with BMI z-score values and FT4 resulted negatively correlated with BMI z-score, demonstrating that THs can influence the individual’s weight even in euthyroid individuals [28–30].

The primary aim of this report was to assess the timing of AR in children with CH, treated regularly with levothyroxine since the first month of life. The analysis revealed that AR appears earlier in the group of children with CH when compared both Italian national and regional (South of Italy) references. These results are coherent with previous published data, despite some variation in the timing of AR. In a retrospective analysis of 53 children with CH, Wong et al. discovered that the timing of AR was earlier in children with CH than in the British reference population, with an inverse relationship between the time of AR and BMI z-score at 10 years (*r* = -0.487,  *p* = 0.01) [12]. Chen S. et al., in a retrospective study involving 90 children with CH, found that girls with CH experienced an AR that was significantly earlier than that of the general population, with a BMI mean value reaching the 97th percentile (3.17 ± 1.38 vs. 3.92 years). Additionally, researchers report that 32.2% of children and adolescents between the ages 6–7 years had obesity or overweight. Early AR, higher BMI value both at the first peak and at AR, and a lower T4 following treatment were associated with obesity at this age [14].

Conversely, Livadas et al. did not observe an early AR in children with CH compared to the reference population. In contrast, no substantial changes in BMI were seen in this group of children after the initial peak during the first year of life, which attenuated the BMI nadir and BMI peak at AR [13].

In our cohort, 30% of the patients were classified as overweight or obese at the age of eight. Furthermore, BMI at eight years of age was significantly correlated with both the age at which AR occurred and the BMI value at the time of AR. Several scientific investigations support this assumption, albeit with varying percentages and ages, and linked early AR to an increased risk of cardiovascular and metabolic consequences in early adulthood, including dyslipidemia, glycemic alteration and arterial hypertension [31–34]. A systematic review by Zhou et al., which included 28 articles up to August 2021, reported a correlation between an early age at AR (age at AR < 5.0-5.1 years) and a higher risk of overweight and obesity from childhood to adulthood. The review also showed that 40% of the individuals assessed experienced an early AR [31]. However, it is important to note that, while our results are consistent with the cited evidence, the 30% prevalence of overweight/obesity in our cohort also reflects the current epidemiological context in Italy. According to the 2023 National Survey on the Weight Status of Children and Adolescents, 19% of Italian children are overweight and 9.8% are obese, with higher prevalence rates were reported in southern regions, the same geographical area from which our sample was drawn [35]. Thus, while our results seem to confirm the association between early AR and an increased risk of overweight/obesity, they may also reflect a trend currently present in the Italian pediatric population, particularly in the South Italy.

No factors associated with early AR were identified in the present cohort of children with CH.

In general, the current evidence suggests that environmental and dietary factors, in addition to genetic factors, play a critical role in explaining the anticipation of this phenomenon. In a report by Goh EK et al., several predictive parameters for the risk of early AR were identified for each period and distinguished by sex, showing that early life diet influence the timing of AR [36]. In a retrospective study of 248 North Carolina children, Ip et al. found that maternal education level, sex, increased BMI, and caloric intake were all potential predictors of early AR [20]. Finally, a French prospective study of 1415 children from the EDEN mother-child cohort study focused on the role of perinatal factors and genetic predisposition to obesity in the timing of AR. Genetic predisposition was assessed using a risk allele score derived from the genotypes of 27 allelic variants identified in genome-wide association studies of adult BMI. This showed that a higher genetic predisposition score for obesity was associated with an earlier age at onset of AR. Maternal and paternal education were positively associated with age at onset of AR, as was parental BMI. Children with a high birth weight had an earlier age at AR, as did children born small for gestational age [37].

This study has some limitations. First is the restricted follow up period, limited to the first 8 years of age, not allowing us to have information on the BMI during the adolescence and the transition age. Another limitation, due to the retrospective design of the study, is the absence of a control group of patients without CH. We adopted the national standards as control group, being aware that these data are not the best representation of the current auxological reality in Italy and that, since the incidence of overweight and obesity is on the rise worldwide, our conclusions will have to be confirmed by new international multicenter studies on large cohorts of patients. Future research involving a larger cohort of subjects with CH with a long-term follow-up is needed to confirm that children with CH have an increased risk of developing childhood obesity and to identify the factors associated with the anticipation of AR. Moreover, a longer follow- up will allow us to evaluate whether a higher prevalence of obesity will also be confirmed in adulthood.

## Conclusions

Children with CH, in this observational study, exhibited an earlier AR compared to the reference population. Early AR may lead to an increased risk of overweight and obesity during childhood and early adulthood. BMI values of these children with CH were higher than those of the Italian national references from early age. The causes of early AR in this population remain unclear, underscoring the need for further future investigations to better understand the contributing factors. For all these motivations, it may be advisable to suggest the implementation of follow-up strategies aimed at promoting a healthy lifestyle, including a balanced diet from birth and regular physical activity in children with CH, who appear to be at greater risk than general population for early AR and its possible long-term consequences [38–40].

## Data Availability

The datasets used and/or analyzed during the current study are available from the corresponding author on reasonable request.

## Abbreviations

AR: Adiposity rebound
TH: Thyroid hormone
BMI: Body mass index
CH: Congenital hypothyroidism
